# Disconnectomics to unravel the network underlying deficits of spatial exploration and attention

**DOI:** 10.1038/s41598-022-26491-6

**Published:** 2022-12-24

**Authors:** Daniel Wiesen, Leonardo Bonilha, Christopher Rorden, Hans-Otto Karnath

**Affiliations:** 1 Centre of Neurology, Division of Neuropsychology, Hertie-Institute for Clinical Brain Research, University of Tübingen, 72076 Tübingen, Germany; 2 Department of Psychology, University of South Carolina, Columbia, USA; 3 Department of Neurology, Emory University, Atlanta, USA

**Keywords:** Cognitive neuroscience, Attention, Perception

## Abstract

Spatial attention and exploration are related to a predominantly right hemispheric network structure. However, the areas of the brain involved and their exact role is still debated. Spatial neglect following right hemispheric stroke lesions has been frequently viewed as a model to study these processes in humans. Previous investigations on the anatomical basis on spatial neglect predominantly focused on focal brain damage and lesion-behaviour mapping analyses. This approach might not be suited to detect remote areas structurally spared but which might contribute to the behavioural deficit. In the present study of a sample of 203 right hemispheric stroke patients, we combined connectome lesion-symptom mapping with multivariate support vector regression to unravel the complex and disconnected network structure in spatial neglect. We delineated three central nodes that were extensively disconnected from other intrahemispheric areas, namely the right superior parietal lobule, the insula, and the temporal pole. Additionally, the analysis allocated central roles within this network to the inferior frontal gyrus (pars triangularis and opercularis), right middle temporal gyrus, right temporal pole and left and right orbitofrontal cortices, including interhemispheric disconnection. Our results suggest that these structures—although not necessarily directly damaged—might play a role within the network underlying spatial neglect in humans.

## Introduction

Spatial neglect is one of the most common behavioural consequences after brain injury affecting predominantly the right hemisphere^1–3^. Spatial neglect affects tasks of spatial attention and orientation^4–6^ and patients typically deviate towards the ipsilesional side, neglecting contralesionally located information or stimuli^7,8^. Crucially, this pathological scene-based (egocentric) spatial bias is not seen with the same frequency or severity following left hemisphere injury^9^. Thus, spatial neglect is thought to be related to lesions affecting brain areas of a right anatomical network, involving frontal, parietal and temporal grey matter areas, and interconnecting white matter fibres^8,10^.

Evidence for a large-scale network in spatial neglect also comes from Parr and Friston^11^, who developed a computational model of attention based on active inference and induced symptoms of spatial neglect by virtually lesioning distinct nodes of this network (i.e. nodes of dorsal and ventral attention networks and contributions of the basal ganglia). Correspondingly, Bogadhi et al.^12^ and Gaffan et al.^13^ found direct causal evidence in the monkey brain, showing that direct and indirect inactivation of crucial hubs of the network produces neglect-like lateralized deficits.

So far, most of the previous anatomical investigations of spatial neglect used methods such as lesion-symptom mapping of stroke survivors^10,13–15^, which focuses on necrotic and gliotic regions from the stroke. However, focal lesions, in addition to local damage, may produce remote dysfunctions due to malperfusion or disconnection in or related to areas that are structurally intact. Disruption of such areas might contribute to the behavioural deficit. In this context, investigations based on fiber tracking^16–22^ or after intraoperative direct electrical stimulation of critical fibers^23^ have highlighted the relevance of specific white matter tracts for spatial neglect. Thus, it appears essential to integrate information about structural disconnection in anatomo-functional models on brain function.

Lesion symptom mapping has several inherent limitations which should be acknowledged (for a comprehensive review, see Karnath et al.^24^), and which promote the use of complementary methods. First, larger injury is likely to result in more impairment: there is more chance of injury to critical module(s) and more chance that enough of the critical module(s) are injured to compromise behaviour. Second, the size and location of injury is not random, but is driven by the vasculature. Therefore, our statistical power is highest in regions that are commonly injured (where we have more observations), and findings may be biased toward the roots of the vasculature^25^, though these biases can be attenuated to some extend^26^. This means that we may be more likely to identify the nodes of distributed functions that are located in regions of common injury. The method we describe here tries to mitigate this effect, by identifying long range connections that are commonly compromised in patients exhibiting an impairment but spared in those without impairment. This can allow us to detect connections where one node may be far from typically injured territory.

Moreover, numerous studies have reported that spatial neglect can be observed after injury that appears restricted to subcortical regions. Several of these studies have found that these individuals also exhibit cortical dysfunction, as observed using measures of blood flow and metabolism^27^. These cases may reflect misery perfusion (where the cortical regions receive enough blood flow to survive, but not enough to function) and diaschisis (dysfunction of distant, but functionally connected brain regions). These consequences cannot be directly observed in structural scans, and at least one caveat of blood flow measures is that they might not be particularly sensitive at detecting the full extent of these effects. As diaschisis is driven by connections between regions, connectome methods like those we employ here may be able to detect regions involved in neglect that are not commonly structurally damaged (e.g. tissue in regions with collateral blood flow that are resilient to direct injury).

In the past, methods for the evaluation of remote structural and functional effects of brain damage in neurological patients have included perfusion weighted imaging (PWI), diffusion tensor imaging (DTI), functional magnetic resonance imaging (fMRI), and resting state functional magnetic resonance imaging (rfMRI) (for an overview, see Karnath et al.^24^).

While rapid perfusion and low-direction diffusion-weighted images are often part of standard diagnostic routines, most of these methods require acquisition of slow research-grade sequences in high-field magnets directly in patient samples. This limits sample size (as participants must endure long procedures), increases cost (as these are not part of standard of care), and reduces the translational potential. Alternatively, one can infer the remote effects of stroke lesions by comparing the location and extent of the injury observed on clinical structural scans and by estimating which long-range connections have been compromised. This approach uses a normative connectome database from existing high-quality research scans acquired in large populations of healthy adults. These connectomes can be based on functional (e.g. resting state connectomes revealing distant hubs that are highly correlated) as well as structural (e.g. diffusion based connectomes of long range structural connections) imaging and lead to the development of new techniques and tools, such as disconnection-symptom mapping (DSM)^28,29^ or connectome lesion-symptom mapping (CLSM)^30,31^. Salvalaggio et al.^32^ found that especially information from such structural disconnection was predictive for most of the behavioural variables investigated, whereas information from indirect functional disconnection (i.e. by evaluation of functional remote dysfunction in normative databases with respect to clinical lesion data) was not. Connectome based lesion-symptom mapping is also especially appealing, as it is the first analysis approach directly linking white matter disruption to grey matter nodes, providing a large scale perspective on structural network organization and dynamics selectively impaired in patient samples^33^.

As discussed in Sperber and Karnath^34^, valid findings employing any lesion-symptom mapping technique require to break down behavioural tasks to cover cognitive functions as isolated as possible. Although spatial neglect patients show pathological behaviour on different scales and there exist several spatial and non-spatial attentional symptoms that have been associated with neglect patients (for review, see Rode et al.^35^), many of these symptoms can dissociate both anatomically and behaviorally and thus we aim to focus only on the egocentric core of spatial neglect (see Karnath and Rorden^8^ as well as Corbetta and Shulman^36^). The latter can be reliably measured by traditional cancellation tasks^37^ as well as a modified line bisection task^38^ and can be defined as the sustained and spontaneous deviation of eyes, head and attentional focus towards the ipsilesional side^39–41^, combined with neglect of contralesionally located information or stimuli.

The present study had two aims. The first was to determine if multivariate connectome-based lesion-symptom mapping is able to predict the severity of pathological behaviour in acute spatial neglect patients. Based on our previous investigation^10^, demonstrating the involvement of multiple areas within a right anatomical network in spatial exploration and attention and specifically in the emergence of the core symptoms of spatial neglect, we hypothesized that multivariate statistical modelling of network disruption—evaluated by fivefold cross-validation—is able to predict pathological behaviour. Moreover, we aimed to evaluate the anatomical topography of disconnections in right brain damaged patients, specifically related to the severity of especially the core egocentric symptoms in spatial neglect. We hypothesized that connectome-based lesion-symptom mapping will allow us to re-evaluate the role of brain areas contributing to behavioural symptoms, but either damaged with less prevalence in unselected stroke populations and/or remotely dysfunctional. This is especially true for structures of the right inferior and middle frontal cortices and the right superior parietal cortex. Whether damage and disconnection to these areas contribute to the development of spatial neglect and/or deficits of spatial attention is still debated. Although functional imaging studies suggest a crucial role of the latter, this cannot be confirmed by investigations employing lesion-symptom mapping in stroke populations with attentional deficits. We expected that information from such an analysis will provide valuable insights into brain functioning, complementing research in the field of lesion-symptom mapping^24^.

## Materials and methods

### Patient recruitment

The sample consisted of 203 neurological subjects admitted to the Centre of Neurology at Tuebingen University, which participated already in a previous investigation^10^. Patients were screened for a first ever right-hemisphere stroke, exclusion criteria included the presence of diffuse or bilateral brain lesions, patients with tumours and patients in whom MRI or CT scans revealed no obvious lesions. Table 1 shows demographic and clinical data. Subjects gave their informed consent to participate in the study, which was performed in accordance with the ethical standards laid down in the revised Declaration of Helsinki and approved by the ethics committee of the medical faculty of Tuebingen University.

**Table 1:** Demographic and clinical data of the 203 patients included.

	No neglect	Neglect
Sex (F/M)	51/71	33/48
Age (years)	60.2 (13.7)	64.7 (12.4)
Visual field defects (% present)	14	27
Time since lesion (days)	3.2 (4.5)	4.2 (4.3)
Imaging (CT/MRI)	53/69	44/37
Etiology (Hemorrhage/Ischemia)	18/104	12/69
Lesion size (cm^3^)	29.3 (35.0)	70.0 (64.4)
Letter cancellation (CoC)	0.01 (0.02)	0.36 (0.32)
Bells cancellation (CoC)	0.01 (0.03)	0.39 (0.29)

For this table we determined whether a CoC (= Center of Cancellation; see Rorden and Karnath^28^) score was in the pathological range; cut-offs were set at > .083 for the Letter Cancellation test and > .081 for the Bells Cancellation Task (cf. Rorden and Karnath^28^. A positive diagnosis of spatial neglect was assigned if a pathological test score in at least one of the two cancellation tests was detectable. 122 (60%) did not exhibit neglect, while 81 (40%) were classified as exhibiting spatial neglect. Data are represented as mean (SD). Note that in the statistical tests we treated the severity as a continuous measure. The table reveals that using cut-off thresholds, there is little variability in patients without pathological bias (ceiling performance). In contrast symptom severity varies across patients with pathological deficits. Modified from Wiesen et al.^10^.

### Neuropsychological examination

Well-established pen and paper tests were used to evaluate the presence of spatial neglect; patients underwent a test routine including the Letter Cancellation Task^42^ and Bells Test^43^. The centre of a 21 × 29.7 cm sheet of paper including the Bells- or Letter-task was aligned with the patient’s sagittal midline. Patients were instructed to mark 60 target letters (‘A’) embedded in a field of distractor letters (for the letter cancellation task) or find 35 bell icons amid a field of distractor icons (for the Bells cancellation task). There was no time limit; patients decided on their own about the end of the tasks. The investigator always asked the patient to confirm twice that they were satisfied with their performance (e.g. initially ‘are you done?’, and ‘are you sure?’ after an affirmative response). To get a stable estimate of the severity of the neglect typical bias, we first calculated the Center of Cancellation (CoC) using the procedure suggested by Rorden and Karnath^37^. The CoC provides a continuous measure for neglect severity, which strongly predicts other popular acute neglect measures. In a final step, we averaged the two cancellation CoC scores to a compound estimate. The interval between stroke-onset and neuropsychological examination was maximally 25 days (mean = 4.37 days, SD = 4.04). Visual field defects were examined by the common neurological confrontation technique.

### Imaging

Structural imaging was acquired either by MRI (n = 106) or CT (n = 97), performed on average 3.5 days (SD = 4.6) after stroke-onset. MR scans were preferred if both imaging modalities were available. In participants where MR scans were available, we used diffusion-weighted imaging (DWI) if the images were acquired within 48 h after stroke onset or T2-weighted fluid attenuated inversion recovery (FLAIR) images for later scans. This mixture of modalities allows to increase sample size and mimics typical standard of care. This variability is orthogonal to our dependent variables and might lead to reduced statistical power but cannot explain positive effects. Further information regarding MRI and CT acquisition parameters can be found in the supplementary material (Table S1). Lesion boundaries were manually marked on the transversal slices of the individual MR or CT scans using the free MRIcron software (www.mccauslandcenter.sc.edu/mricro/mricron). Normalization of CT or MR scans to MNI space with 1 × 1 × 1 mm resolution was performed by using the Clinical Toolbox^44^ under SPM8 (www.fil.ion.ucl.ac.uk/spm), and by registering lesions to its age-specific templates oriented in MNI space for both CT and MR scans^44^. If available, MR scans were co-registered with a high resolution T1-weighted structural scan in the normalization process. Delineation of lesion borders and quality of normalization were verified by consensus of always two experienced investigators (one of them H.-O.K.). An overlap of all normalized lesions is shown in the supplementary material (Fig. S1). The average lesion size in the sample was 45.52 cm^3^ (SD = 52.67 cm^3^). In the supplementary material of the previous investigation^10^, we show overlap plots of normalized lesions separated for each imaging modality (Fig. S1) as well as a histogram of the lesion size distribution (Fig. S2B). Moreover, we provide a figure showing the regional bias caused by lesion volume (Fig. S3) in the same published work.

### Preprocessing of the link-wise structural (dis)connectome

To evaluate structural disconnection, we calculated the white matter link-wise disconnectome, quantifying the disconnection between any two gray matter Regions of Interest (ROIs) based on individual lesions. The ROIs were derived based on gray matter areas parcellated in the Desikan–Killiany atlas^45^, provided by the IIT Human Brain Atlas (v.5.0) (https://www.nitrc.org/projects/iit/; Zhang and Arfanakis^46^). All 84 ROIs were included.

In a first step, we created a whole brain tractogram. This was based on a template of healthy subjects (i.e. normative data of 72 healthy human subjects; 28 male, 29 ± 6 years of age, 20–40 years of age). For full information regarding acquisition parameters and details on the construction of this template, we refer to the original work of Varentsova et al.^47^. We used the Spherical harmonic (SH) coefficients of the provided HARDI template (*IIT_HARDI.nii*), including fibre orientation distributions (FOD). All fibre tractography steps were carried out by the tractography package MRtrix3 (https://www.mrtrix.org/)^48^. To create the whole brain tractogram, we first prepared a seeding mask for performing anatomically constrained tractography (ACT)^49^ by using the *5tt2gmwmi* command in MRtrix3 on the 5 tissue type segmented anatomical image (*IIT_fornix_fixed_5tt_file_for_ACT_tractography.nii*), which was provided by the IIT Human Brain Atlas (v.5.0). This increases the biological plausibility of downstream streamline creation by improving the accurate determination of where streamlines should be terminated^49^. Then, we generated streamlines using the *tckgen* command with the default *Second-order Integration over Fiber Orientation Distributions* (*iFOD2*)^50^ probabilistic algorithm, anatomical constrained deconvolution, seeding 10 million streamlines at the grey matter–white matter boundary and by using *backtrack*^49^ to allow tracks to be truncated and re-tracked if a poor structural termination is encountered. Next, we down-filtered the tractogram from 10 to ~ 1.5 million streamlines, such that the streamline densities match the FOD lobe integrals, by using the *Spherical-Deconvolution Informed Filtering of Tracks *(*SIFT*)^51^ algorithm. This reduces the constrained spherical deconvolution-based bias in overestimation of longer tracks compared to shorter tracks. In general, by using this filtering procedure, the number of streamlines connecting two regions becomes a proportional estimate of the cross-sectional area of the fibres connecting those two regions, increasing again the biological accuracy of the tractogram. In a recent study from Yeh and et al.^52^, the authors demonstrate that streamline count becomes a valid biological marker of connection density after using ACT and SIFT together. Consequently, we refrained from additionally applying a popular scaling mechanism often used in the field to control for false positive streamlines after probabilistic tractography (i.e. scaling by the inverse streamline length). Hence, this scaling mechanism provides only an incomplete correction for the biases targeted (in comparison to the application of ACT and SIFT)^52^.

In a second step, we estimated the ‘healthy’ normative connectome based on the whole brain tractogram from the previous step with respect to the cortical parcellation of the atlas file (*IIT_GM_Desikan_atlas.nii*). All subsequent preprocessing steps were carried out with MATLAB 2018b. We read in the coordinates of each individual tract into a vector and quantified for any two ROI’s of the parcellation the number of tracks connecting them. As we performed fibre tracking by using ACT, the precise location of streamline termination did not automatically overlap perfectly with the borders of the parcellation of the Desikan atlas^45^. Hence, a radial search of 2 mm radius was performed at each streamline start and termination point, to find the nearest gray matter label. Although we ensured that streamlines start and end at the GM/WM boundaries and connecting one area to another, we further controlled for any inaccuracies during tract reconstruction and discarded streamlines running through 3 or more ROI’s from the tractogram, before deriving the connectome. Although it is often recommended to scale the links between any two ROI’s by the inverse of their size, we decided to not perform such a control. Indeed, larger ROI’s tend to be intersected by a larger number of streamlines as smaller ROI’s. This is not based on a higher connectivity strength, but on a physically larger ROI with an increased likelihood of a larger number of fibers entering/exiting the white matter. We consider this, however, as an interesting feature of the network, rather than an effect that should be eliminated. Moreover, scaling the links by the inverse of the ROI volumes might have impacts on the intuitive interpretability of statistical outcomes, as we are not looking any more at the strength between any to links and their relation to pathological behaviour but at the strength between any to links scaled by ROI volumes and behavioural findings. Finally, in the present study, the correlation of streamline counts between any two ROI’s of the connectivity matrix and their mean volume is only small (r = 0.04) and thus make the latter scaling mechanism dispensable.

We then registered each lesion to the stereotaxic atlas space, discarded for each patient tracks running through the lesion and repeated the above procedure for connectome generation by creating for each patient a connectome matrix with spared ROI-to-ROI connections. As we were especially interested in disconnected links, we subtracted each link of the ‘healthy’ normative whole brain connectome file by the corresponding link of the respective spared patient connectome, extracting an 84 × 84 matrix per patient reflecting the link-wise (i.e., ROI-to-ROI) disconnectome. In practical terms, this corresponds to the number of tracks running through any two Desikan atlas ROI’s and disconnected by the lesion. From this matrix, we extracted a subnetwork retaining only links altered in at least one patient and discarded reciprocal connections. For each patient a row vector of right intrahemispheric and interhemispheric links was then used for the subsequent multivariate connectome lesion-symptom mapping analysis.

### SVR-CLSM analysis

Our statistical analysis was performed with a multivariate method that recently gained popularity in the field of lesion-symptom mapping, namely SVR-LSM (Support Vector Regression based Lesion Symptom Mapping)^53^. Support vector regression is a supervised machine learning technique^54,55^ which is able to model the continuous relationship between lesion data and behavioural scores. This method was used and validated successfully in previous investigations with real lesion-symptom data^10,56^ or synthetic features^57^. For a detailed description about this method, we refer to the original study by Zhang et al.^53^. Using this same procedure, but with disconnectome matrices instead of traditional lesion maps, we performed support vector regression-based connectome lesion-symptom mapping (SVR-CLSM).

All the analyses were performed with MATLAB 2018b and libSVM 3.23^58^, using a linear Kernel. A parameter optimisation procedure via grid search was carried out with fivefold cross-validations to find the C model parameter (search range of C = 2^–30^–2^30^ in steps of 1) with the best trade-off between prediction accuracy and reproducibility, similar to the procedure of a previous investigation^28^. In the present experiment, the optimisation procedure resulted in empirically optimised values for the model parameter C = 2^–17^. The optimised C was then used in the final SVR-CLSM analysis. During this analysis, we derive SVR *β*-parameters for each voxel as described by Zhang et al.^53^, reflecting the model weight (i.e. strength) between structural disconnection of any two ROIs and the mean CoC score. These *β*-parameters were then tested in a link-wise permutation algorithm to assess statistical significance based on 10.000 permutations. During this procedure the SVR *β*-parameters for each link are compared with new *β*-parameters drawn for each permutation through randomisation of behavioural scores, resulting in a link-wise topographical probability map. Results are reported at *p* < 0.05 after Bonferroni correction for multiple comparisons. As statistical testing is performed for each individual link, a form of multiple comparison correction is required to prevent an increase of false alarms^57^. Significant links were interpreted and labelled according to the parcellated ROI’s of the Desikan–Killiany atlas^45^.

### Ethical approval

Subjects gave their informed consent to participate in the study, which was performed in accordance with the ethical standards laid down in the revised Declaration of Helsinki and with local guidelines and regulations of the University Hospital Tuebingen.

## Results

### Parameter optimization

To find the best *C* parameter for the SVR-CLSM analysis, we ran a 5 times fivefold cross-validation routine. This resulted in an average prediction accuracy of *r* = 0.46 (*R*^2^ = 0.21) with *C* = 2^–17^, which was used in the final analysis.

### Structural (dis)connectome analysis

**Figure 1:**
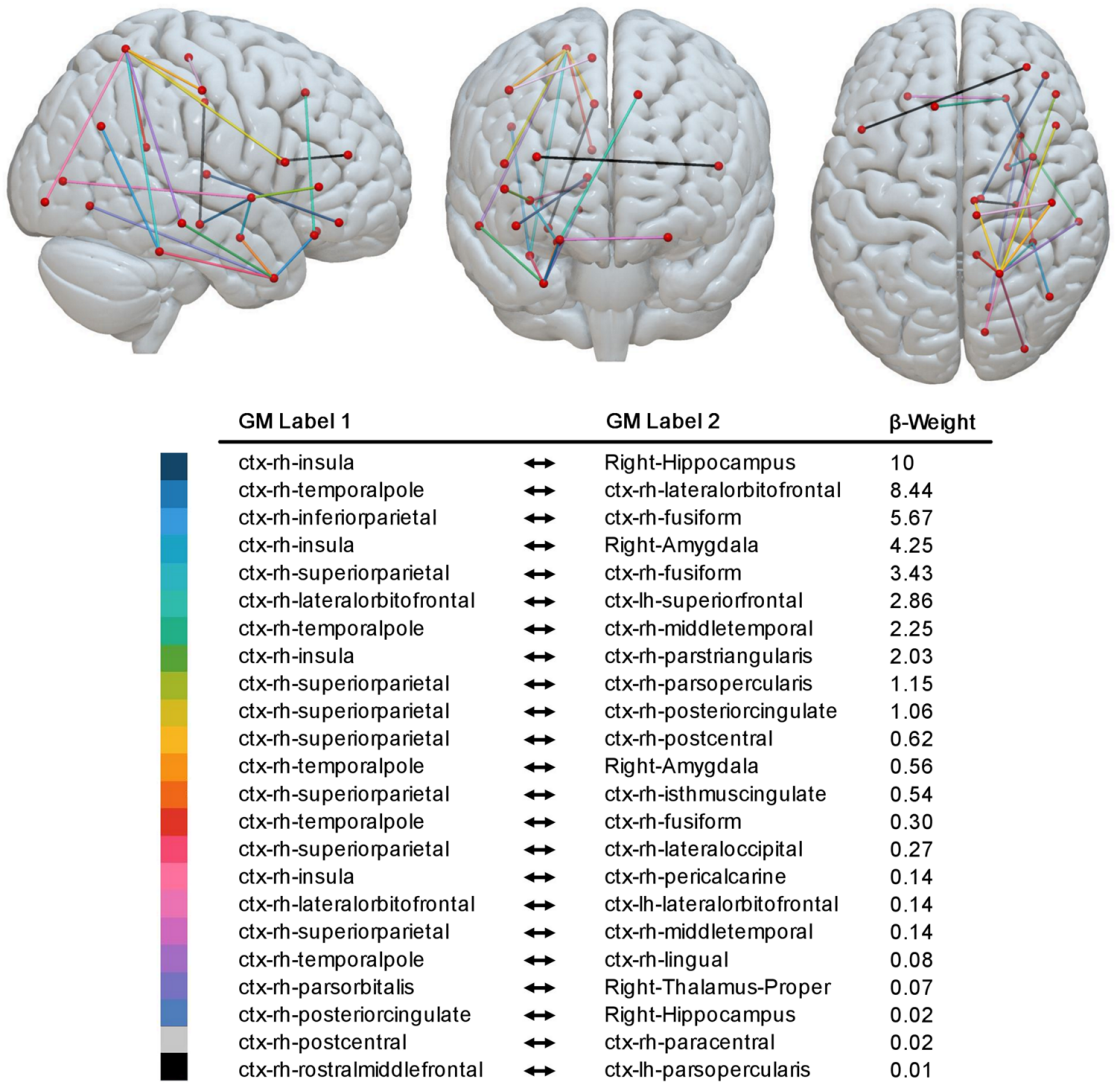
Results of the SVR-CLSM analysis. Support vector regression based connectome lesion-symptom mapping results using data of 203 patients. Link-based probability-map of SVR-CLSM on CoC scores after 10.000 permutations, shown at *p* < 0.05 (Bonferroni corrected for multiple comparisons) illustrating anatomical regions disconnected to other brain regions which are related to the core deficit of spatial neglect. Each color is associated to one disconnected link. The (dis)connectome is shown from sagittal view (right hemisphere), frontal view and superior view. The table further provides a *β*-weight per link. Parcellation into grey matter connectome nodes was done with respect to the Desikan–Killiany atlas^45^.

**Figure 2:**
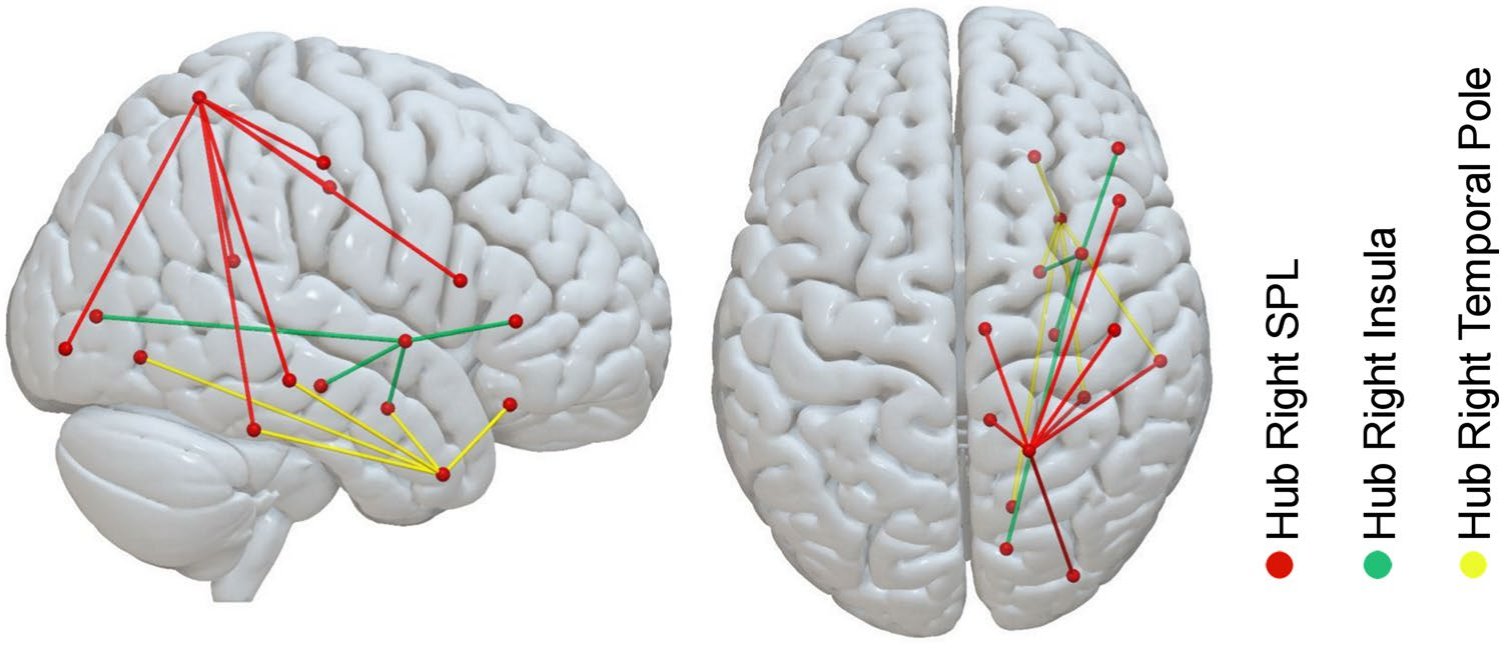
Results of the SVR-CLSM analysis reduced to crucial hubs. To better illustrate findings from Fig. 1, we segmented the (dis)connectome map into subnetworks, depending on whether there were continuous links between nodes or not. We extracted four different components. The figure shows disconnections of the three main hubs with continuously disconnected links of component one. Each color is associated to the disconnected edges of one of the three major hubs with more than three disconnections (the right insula, the right SPL and the right temporal pole). Results are shown from sagittal view (right hemisphere) and superior view. Parcellation into grey matter connectome nodes was done with respect to the Desikan–Killiany atlas^45^. *SPL* superior parietal lobule.

Figure 1 shows all disconnected links significantly related to higher CoC scores (our measure of neglect severity). To facilitate interpretation of the results, we segmented the resulting disconnection network map into subnetworks, depending on whether there were continuous links between nodes or not. Within the first component, we extracted three main right hemispheric hubs with disconnections to > 3 other areas of the brain (cf. Fig. 2), the right superior parietal lobule (7 ROI-to-ROI disconnections), the right insula (4 ROI-to-ROI disconnections), and the right temporal pole (5 ROI-to-ROI disconnections). Although we evaluated only direct links (i.e. no secondary or tertiary disconnections), it is important to note, that the disconnection profile of all damaged links as a whole (i.e. whole network) contributed to the prediction of the behavioural symptom.

Within the first component (cf. Fig. 2), we found in patients with higher impairment, i.e. higher CoC scores, disconnection in the right hemisphere between the superior parietal lobule (SPL) and the fusiform gyrus, SPL and the inferior frontal gyrus (pars opercularis), SPL and the posterior part of the cingulate cortex, SPL and the isthmus of the cingulate cortex, SPL and the lateral area of the occipital gyrus and between SPL and the middle temporal gyrus. A second important hub within the first component was identified as the right insula. In patients with higher impairment (higher CoC scores), we found higher inter-regional disconnection in the right hemisphere between insula and hippocampus, insula and amygdala, insula and the inferior frontal gyrus (pars triangularis), and between insula and the pericalcarine area in the occipital lobe. The third hub of the first component constituted the right temporal pole (TP) with inter-regional disconnections specifically between the TP and lateral orbito-frontal cortex, TP and middle temporal gyrus, TP and amygdala, TP and fusiform gyrus, and between the TP and the lingual gyrus. Within the first component, additional structural disconnection between the right fusiform gyrus and the right inferior parietal gyrus, the right posterior cingulate and right hippocampus, the lateral part of the right orbito-frontal cortex and the left superior frontal gyrus and between the lateral part of the right orbito-frontal cortex and the lateral part of the left orbito-frontal cortex were related to higher CoC scores.

Components two, three and four were characterized by single ROI-to-ROI disconnections (i.e. structurally not related to other areas of the brain in our analysis) associated to spatial neglect. Component two shows that the link between the right thalamus and the right inferior frontal gyrus (pars orbitalis) was affected. Component three shows that disconnection between the right postcentral gyrus and right paracentral gyrus might be important. Finally, component four identified an interhemispheric disconnection between the rostral section of the right middle frontal gyrus and the left inferior frontal gyrus (pars opercularis).

## Discussion

The goals of the present investigation were to test the hypotheses that (1) network damage leads to spatial neglect and can predict the severity of pathological behaviour and that (2) connectome lesion-symptom mapping is able to refine topographical maps of anatomical damage leading to disorders of spatial attention and neglect by allowing the statistical evaluation of remote structural dysfunction. Therefore, we localised remote structural disconnection and central hubs beyond the typical territorial affection in right brain damaged patients.

We combined a multivariate analysis technique based on support vector regression with connectome lesion-symptom mapping (SVR-CLSM) to predict the severity of spatial neglect. The predictive performance of our model was comparable to a former investigation using the same dataset, but using only information form direct anatomical damage (i.e. R^2^ = 0.19)^10^. Furthermore, we detected three central nodes, namely the right superior parietal lobule, the right insula and the right temporal pole, which were extensively disconnected from other intrahemispheric areas. Moreover, central links to ventral prefrontal areas (i.e. inferior frontal gyrus and orbitofrontal cortex) and to the middle temporal gyrus were found within the network structure. Our results suggest that these areas might play a role within the anatomical network underlying spatial neglect.

### Superior parietal lobule

Following an influential anatomo-functional model of visuospatial attention^5,36^, the superior parietal lobule and intraparietal sulcus (SPL/IPS) were considered to be part of a dorsal attention network (DAN) showing increased BOLD activity in controlled goal directed attention towards visual targets, reflecting endogenous attentional processing. In combination with the ventral attention network (VAN), reflecting exogeneous attentional capabilities, these two networks were assumed to contribute to normal visuospatial attention. Beyond, the authors suggested that disconnection of pathways of this network may play an important role in the development of spatial neglect^4,36,59^. In particular, they argued that structural damage to the VAN produces a functional alteration of the DAN—in the SPL/IPS—and that this structural–functional linkage is the main mechanism evoking the behavioural disorder.

In line with this model, our present SVR-CLSM analysis revealed the SPL as one of the three major hubs in which disconnection correlated with neglect severity. The analysis detected a disruption between the SPL and the inferior frontal gyrus (pars opercularis) in the ventral prefrontal cortex (VFC). The evaluation of functional imaging findings from He et al.^59^ in patients and healthy subjects together with findings of lesion-symptom mapping studies in neglect patients^16,22,60,61^, lead to the suggestion that areas within the VFC—i.e. mainly the inferior and middle frontal gyri—might serve as critical coordination nodes between brain systems for endogenous (DAN) and exogeneous (VAN) spatial attention^62^. Following Hattori et al.^62^, projections of the inferior-frontal occipital fasciculus (IFOF) link superior parietal areas of the DAN (e.g. SPL, IPS) with areas of the VFC^63,64^. Accordingly, lesion studies showed that direct damage to the IFOF can lead to the development of spatial neglect^10,16,20,65^. Our present results might indicate that communication between DAN and VAN in spatial neglect patients is not only altered after damage to projections of the second branch of the superior longitudinal fasciculus (SLF II), as shown previously^17,18,23^, but also by projections of the IFOF (i.e. disconnecting superior parietal and ventral frontal areas).

Despite these numerous findings, the functional model of visuospatial attention as a comprehensive explanation for the development of spatial neglect was also criticised. The remote functional alteration of the SPL (evoked by a structural lesion of the VAN), leading to an interhemispheric imbalance between right and left SPL, i.e. left and right DAN function^4,36^ was also observed in further neurological samples. For example, Umarova et al.^66^ showed that an abnormal interhemispheric imbalance pattern between right and left parietal areas occurs independently of a positive neglect diagnosis after right hemispheric stroke and after using a similar ‘spatial attention’ paradigm as in Corbetta et al.^4^. Moreover, de Haan et al.^67^ evaluated the BOLD signal in acute stroke patients without neglect and observed an abnormal interhemispheric balance consisting of reduced signal change in perilesional areas of the damaged hemisphere relative to homologous areas in neurologically healthy controls, unrelated to the patients’ behaviour. This data appears to suggest that abnormal interhemispheric imbalance after stroke—such as the one reported by Corbetta et al.^4^ may often merely reflect a decoupling of the neurovascular response without changes in neuronal functioning and/or in the individuals’ behaviour, or a combination of these two effects. Taking these findings together, the abnormal interhemispheric imbalance in the SPL/IPS region after damage in the VAN system might reflect a physiological epiphenomenon, for example, cerebrovascular reactivity^68–70^ (see for discussion Karnath et al.^6^), and not necessarily a change in neural function related to the behavioural defects in spatial neglect.

Moreover, there is a noticeable discrepancy in findings between lesion studies in neglect patients and studies using fMRI to investigate spatial attention in unimpaired subjects (as noted by Szczepanski et al.^71^). Structural lesion studies in stroke patient samples typically provide sparse evidence for a distinct role of the SPL in spatial neglect. To date, there are only few reports in patient studies devoting a role to the SPL/IPS complex in the occurrence of spatial neglect^61,72–75^. Vice versa, it was reported that patients with lesions affecting SPL/IPS in particular, show attentional disorders but no spatial neglect. For example, Gillebert et al.^75^ demonstrated in two patients with small focal lesions (one with right SPL/IPS damage, the other with left IPS damage; in both cases the inferior parietal lobule was spared) that damage of the SPL or IPS led to lateralised deficits in a spatial attention paradigm which were similar to those observed in a control group of patients with focal IPL damage. Specifically, these patients were not able to reorient their attention to and to select between competing stimuli for contralesional targets. Accordingly, Vandenberghe et al.^76^ noted that damage to the SPL or the IPS might generate a general spatial attention bias, occurring after right or left hemisphere damage. However, like Gillebert et al.^75^, they also noted that none of the patients with isolated SPL damage they discussed had clear signs of spatial neglect (no pathological performance in e.g. a star cancellation task). These observations suggest that behavioural deficits observed after SPL/IPS damage are not spatial neglect specific. Evidence for this comes from Rorden and Karnath^77^, who describe how more posterior injury is associated with the line bisection task, a task which shows poor correlation with other neglect symptoms^78^. Rather, it appears that spatial neglect—at least the egocentric core symptoms as defined by Corbetta and Shulman^36^ and Karnath and Rorden^8^—may be guided by mechanisms which are not strictly attentional.

On this background, Karnath^6^ proposed that the top-down control of spatial attention (i.e. the voluntary shifts of spatial attention) originating in the DAN system may actually be spared in neglect patients and that the disorder of spatial neglect is not based on a deficit in top down control of spatial attention. Rather, patients with spatial neglect could suffer from an altered representation of own body position with respect to external objects, based on a biased spatial transformation matrix. In other words, the default centre used to initiate voluntary shifts of spatial attention is biased, but not the voluntary shifts of spatial attention itself. In fact, when neglect patients explore the surroundings by overt shifts of attention, they voluntarily execute movements into all possible directions without obvious direction-specific disturbances^79,80^. The biased distribution of exploratory activity is assumed to result from an altered representation of own body position with respect to external objects. In neglect patients, this body-centred matrix (or egocentric reference frame of topographical information) is assumed to be rotated towards a new ‘default position’ on the right^6,81^. In line with this view, studies showed that the biased exploration centre in neglect patients can be reweighted again to a normal ‘position’ by the systematic manipulation of afferent information, e.g., vestibular or neck-proprioceptive stimulation^82,83^.

### Insula

The second crucial hub in our analysis whose disruption to other brain regions contributed to the severity of spatial neglect, was the right insula. Lesion of this area was found to be related to spatial neglect in many previous lesion-behaviour mapping studies^60,65,84–89^ and two ALE meta-analyses^90,91^. In apparent contradiction, Corbetta and Schulman^36^ suggested, that the insula as part of the VAN contributes mainly to non-spatial attention, for example, arousal and vigilance or the discrimination between relevant or novel stimuli. Indeed, beyond the hallmark spatial bias of exploration and attention, patients with spatial neglect also show many non-spatial attention deficits, which can further exaggerate the neglect-typical spatial biases^92–98^. Together with the dorsolateral anterior cingulate cortex, the amygdala and other subcortical structures, the right insula is also often grouped as ‘salience network’ (for a discussion see Uddin et al.^99^. The damage to the link between right insula and amygdala observed in the present study could indicate that this ‘salience network’ is disturbed in patients with spatial neglect.

Nevertheless, damage to the insula and associated white matter fibres could also reflect an epiphenomenon in spatial neglect patients and represent a direct consequence of MCA territory infarction and its large representation in typical spatial neglect patient samples. At first sight evidence for this notion comes from a recent investigation by Smith et al.^100^. The authors informed a machine-learning based classifier with ROI based lesion information to separate neglect patients from other right brain damaged patients and found, for instance, that damage to the insula alone does not have a unique predictive weight. However, combined with lesion information from other ROI’s, the classification performance of the insula improved considerably. With respect to the present findings, it is thus likely, that specifically the disconnection of the insula to other areas of the brain is crucial in the development of spatial neglect behaviour and that damage to the insula alone is not sufficient.

### Temporal pole and the middle temporal gyrus

The temporal pole (TP) was identified as a third important hub in our analysis. It is an area of the brain rarely reported in lesion-behaviour mapping studies of spatial neglect. In Wiesen et al.^10^, the TP directly contributed to the prediction of the severity of spatial neglect in the acute phase of the stroke. In a sample of 140 acute right brain damaged patients, Smith et al.^100^ showed that power to predict spatial neglect was significantly increased when the TP was added to the pars triangularis of the inferior frontal gyrus and the supramarginal gyrus. Further, in a longitudinal study of 54 patients with right hemisphere damage, Karnath et al.^65^ found lesions of the TP to predict the persistence of spatial neglect symptoms in the chronic phase of the stroke. In line with these previous findings, our present analysis revealed that disconnections between the TP and parts of the orbitofrontal cortex contributes to neglect severity. The disconnection thus might reflect a disruption of the u-shaped uncinate fasciculus, connecting the TP to the VPC but also to the amygdala^101^. Nevertheless, due to the rare reports so far for TP involvement in the emergence of spatial neglect, the exact role needs to be clarified in future works.

Interestingly, our results also depicted the right middle temporal gyrus (MTG) as disconnected to the SPL and TP in contributing to neglect severity. As shown in several previous lesion mapping studies, damage to the MTG seems to play an important role in spatial neglect^10,22,60,65,84,89,102,103^. An important role of temporal structures in spatial neglect and covert attentional processing was also demonstrated in a recent investigation in the monkey brain^12^. This pioneering study showed how focal lesions affect whole attentional circuits in the brain and that pathological behaviour might not always be the consequence of damage to one exclusive brain node or connection. The authors temporarily inactivated an area at the intersection between the posterior middle and superior temporal gyri (posterior superior temporal sulcus [STS]) and observed spatial neglect-like symptoms in the monkeys’ behaviour. They noted that this area in the temporal cortex might be a shared node between DAN and VAN. It is possible that the MTG node we identified in the present study actually relates to the STS. Unfortunately, we could not test this directly. In order to generate the healthy connectome in our analysis, we used pre-processed files of the IIT Human brain atlas using a gyral-based cortical parcellation^45^. Therefore, our analysis is restricted to this parcellation scheme, which does not account for the sulci in the brain.

### Interhemispheric callosal connection

It was suggested that interhemispheric callosal—especially splenial—disconnection might contribute to the development and persistence of spatial neglect^104–109^. In contrast, our analysis revealed no disrupted links between left and right posterior brain areas as anatomical markers for spatial neglect. Rather, we observed that a link between left and right orbitofrontal cortices was disconnected and related to the disorder. The latter is in line with findings from Lunven et al.^110^, suggesting a role of frontal rather than parietal interhemispheric damage in spatial neglect and specifically a failure of rehabilitation by prism glasses.

### Conclusion and perspective

The connectome lesion-symptom mapping approach combined with machine-learning based statistical analysis allowed us to point out a complex network structure in patients with spatial neglect. Our findings underline the importance of a right hemispheric network structure in spatial exploration and attention with a few central hubs largely disconnected from other areas of the brain. The present analysis and findings indicate that traditional lesion-behaviour mapping may be complemented by additional analysis techniques, approaching step-by-step the high-dimensionality in lesion data. A remaining task for future studies is to analyse the specific contribution of each of these network nodes to the development of the behavioural disorder. Further, it is necessary to integrate these findings into current theoretical models about the pathophysiological mechanisms of spatial neglect as well as in models of normal processes of spatial exploration and attention.

## Supplementary Information

Supplementary Information.

## Data Availability

The datasets generated and analyzed during the current study are not publicly available due to the data protection agreement of the Centre of Neurology at Tübingen University, as approved by the local ethics committee and signed by the participants. We provide the custom code of the main analyses, the statistical topographies and overlap maps, available at 10.17632/jgnpbpbbx5.2.

## Abbreviations

ACT: Anatomical constrained tractography
CoC: Center of Cancellation
DAN: Dorsal attention network
DSM: Disconnection symptom mapping
FOD: Fibre orientation distribution
HARDI: High angular resolution diffusion imaging
IFOF: Inferior-frontal occipital fasciculus
IPS: Intraparietal sulcus
MTG: Middle temporal gyrus
SLF: Superior longitudinal fascicle
SPL: Superior parietal lobe
STS: Superior temporal sulcus
SVR: Support vector regression
SVR-CLSM: Support vector regression-based connectome lesion-symptom mapping
SVR-LSM: Support vector regression based lesion symptom mapping
TP: Temporal pole
VAN: Ventral attention network
