# Supplementary material for: Disconnectomics to unravel the network underlying deficits of spatial exploration and attention
Source: Sci Rep. 2022 Dec 24;12:22315. doi: 10.1038/s41598-022-26491-6 (PMC9789971; doi:10.1038/s41598-022-26491-6)
Supplement: Supplementary file 1 — Supplementary Information. [file 41598_2022_26491_MOESM1_ESM.doc]

**Disconnectomics to unravel the network underlying deficits of spatial exploration and attention**

Daniel Wiesen^1^*, Leonardo Bonilha^3^, Christopher Rorden^2^, Hans-Otto Karnath^1,2^

**Supplementary Information**

**
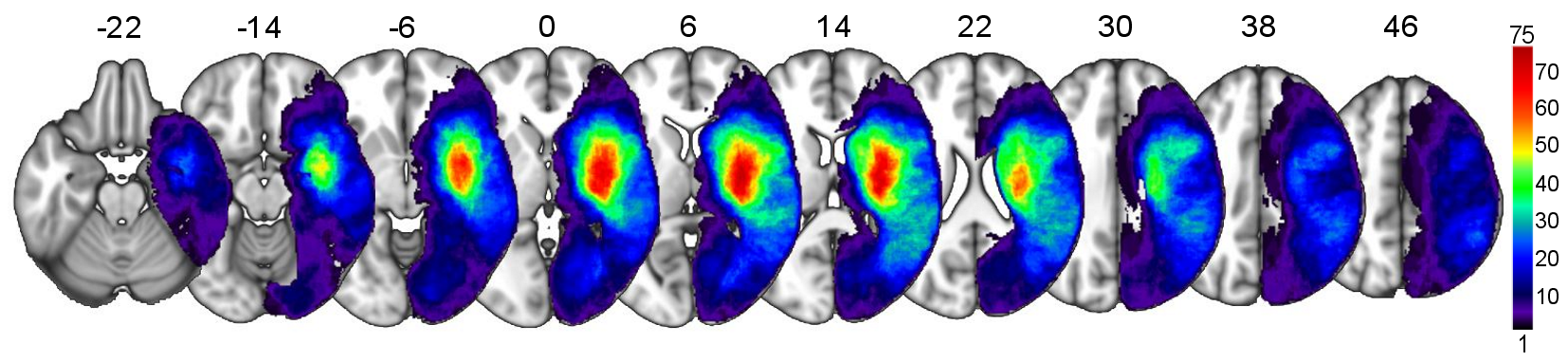
**

**Figure S1: Topography of brain lesions**

Lesion overlap topography of the lesions of all 203 patients. The colorbar indicates the number of overlapping lesions with peak at N = 75. Numbers above the slices indicate z-coordinates in MNI space.

**Table S1**

**Overview of CT and MRI acquisition parameters**

The table shows a range of typical acquisition parameters employed in standard clinical neuroimaging at the University Hospital Tübingen to identify the extend and location of brain damage after stroke in the participants included in the present study. For the most part, images collected with these protocols were used to delineate lesions in our sample of 203 patients, variations of the parameters were possible based on clinical decisions.

| **Parameter MRI/CT Imaging** | **MRI - T2 FLAIR** |
| --- | --- |
| Scanner Types used | Siemens Magnetom Sonata; Siemens Magnetom Aera; Siemens Magnetom Avanto; Magnetom Prisma fit |
| Repetition time (ms) | 8800-9330 |
| TE (ms) | 118-122 |
| Inversion Time (ms) | 2500 |
| Flip Angle | 180 |
| Slice thickness (mm) | 2-5 |
| Slice spacing (mm) | 4-4.4 |
| Voxel size (mm³) | 0.86-1 x 0.86-1 x 2-5 |
| **Parameter MRI/CT Imaging** | **MRI - DWI** |
| Scanner Types used | Siemens Magnetom Sonata; Siemens Magnetom Aera; Siemens Magnetom Avanto ; Magnetom Prisma fit |
| Repetition time (ms) | 3200-4100 |
| TE (ms) | 87-88 |
| Flip Angle | 90 |
| Slice thickness (mm) | 4-5 |
| Slice spacing (mm) | 4-6 |
| b-value | 0-1000 s/mm2 |
| Voxel size (mm³) | 0.89 x 0.89 x 4-5 |
| **Parameter MRI/CT Imaging** | **CT** |
| Scanner Types used | Siemens Somatom Sensation 16; Siemens Somatom Definition AS |
| Number of slices | 35-46 |
| Slice thickness (mm) | 4-4.5 |
| In-Plane resolution (mm) | 0.39-0.52 x 0.39-0.52 |
| Beam energy | 100-140 kVp |
| Tube current | 212-285 mA |
| Reconstruction diameter (mm) | 200-220 |
| Focal spot size (cm) | 1.2 |

**Additional information regarding imaging data used in the present study:**

In our previous work (Wiesen, D., Sperber, C., Yourganov, G., Rorden, C. & Karnath, H.-O. Using machine learning-based lesion behavior mapping to identify anatomical networks of cognitive dysfunction: Spatial neglect and attention. *Neuroimage* **201**, 116000, [2019]), where we used the same dataset, we provided overlap plots of all normalized lesions separated for each imaging modality (Figure S1) together with a histogram of the lesion size distribution (Figure S2 B), as well as a figure showing the regional bias caused by lesion volume (Figure S3).
